# Synthesis and characterization of mesoporous bioactive glass nanoparticles loaded with peganum harmala for bone tissue engineering

**DOI:** 10.1016/j.heliyon.2023.e21636

**Published:** 2023-11-02

**Authors:** Maria Bibi, Syeda Ammara Batool, Sajid Iqbal, Shaher Bano Zaidi, Rabia Hussain, Memoona Akhtar, Ahmad Khan, Mohammed S. Alqahtani, Mohamed Abbas, Muhammad Atiq Ur Rehman

**Affiliations:** a Department of Materials Science and Engineering, Institute of Space Technology Islamabad, Islamabad 44000, Pakistan; b Department of Nuclear and Quantum Engineering Korea Advanced Institute of Science and Technology (KAIST) 34141, Daejeon, Republic of Korea; c Radiological Sciences Department, College of Applied Medical Sciences, King Khalid University, Abha 61421, Saudi Arabia; d Electrical Engineering Department, College of Engineering, King Khalid University, Abha 61421, Saudi Arabia

**Keywords:** Peganum harmala, Mesoporous bioactive glass nanoparticles, Osteomyelitis, Antibacterial, Bioactivity

## Abstract

Globally, there is an increase in a number of bone disorders including osteoarthritis (OA), osteomyelitis, bone cancer, and etc., which has led to a demand for bone tissue regeneration. In order to take use of the osteogenic potential of natural herbs, mesoporous bioactive glass nanoparticles (MBGNs) have the ability to deliver therapeutically active chemicals locally. MBGNs influence bioactivity and osteointegration of materials making them suitable for bone tissue engineering (BTE). In the present study, we developed *Peganum Harmala* (*P. harmala*) loaded MBGNs (PH-MBGNs) synthesized via modified Stöber process. The MBGNs were analyzed in terms of surface morphology, chemical make-up, amorphous nature, chemical interaction, pore size, and surface area before and after loading with *P. harmala*. A burst release of drug from PH-MBGNs was observed within 8 h immersion in phosphate buffer saline (PBS). PH-MBGNs effectively prevented *Staphylococcus aureus* (*S. aureus*) and *Escherichia coli (E. coli*) from spreading. Furthermore, PH-MBGNs developed a hydroxyapatite (HA) layer in the presence of simulated body fluid (SBF) after 21 days, which confirmed the *in-vitro* bioactivity of MBGNs. In conclusion, PH-MBGNs synthesized in this work are potential candidate for scaffolding or a constituent in the coatings for BTE applications.

## 1 Introduction

The high rate of bone disorders and fractures due to accidents has drawn broad investigations into bone tissue recovery. The number of adults over the age of 50 diagnosed with different musculoskeletal disorders including osteoporosis, osteoarthritis, osteomyelitis, and bone cancer is increasing day by day [1,2]. Some diseases including osteomyelitis are caused by bacterial infection. Most instances of osteomyelitis are brought about by kinds of microbes generally found on the skin or in the nose of even healthy people, for instance, staphylococcus aureus (*S. aureus*). The well-known treatment for osteomyelitis is a medical procedure to eliminate parts of the bone that are infected or dead, followed by intravenous anti-microbial given in the clinic [3,4].

Bone tissue engineering (BTE) is a multidisciplinary field, giving an advanced and effective solution for bone repair and regeneration. Several strategies are being implemented currently in bone-related surgeries. Local delivery of bioactive substances added to bone substitute materials can help in fast bone regeneration [5,6]. For example, bioactive glasses (BG), and calcium phosphates (CaPs) are getting extensive innovative research interest for BTE due to their excellent bioactivity, osteoinductivity, osteoconductive, and degradability [7].

The BGs’ tendency of bone-bonding in the presence of physiological fluids is referred to the formation of a hydroxyapatite (HA) layer on their surface. The chemical composition if this layer matches to the mineral phase of natural bone; therefore, it is not recognized by the body as a foreign object [8,9].

Recently, mesoporous BG nanoparticles (MBGNs) have gained significant attention in the bone tissue regeneration application. MBGNs offer application in BTE and controlled drugs release due to their porous surface morphology [10]. The porosity in MBGNs provides adequate space for drug loading that can promote contact area of bioactive material with surrounding tissues [11,12]. MBGNs have shown aptitude for the on-site delivery of therapeutic metal ions [13]. Distinguished therapeutic ions may impact bone recovery either straightforwardly, e.g., such as calcium's stimulation of calcification, or in implication, e.g., by working on the angiogenic properties of therapeutic ions like manganese, strontium, and copper. Properties of MBGNs can be modified by the functionalization of MBGNs via doping of ions or compounds due to the nano porosity resulting from the synthesis process. Different techniques are used to synthesize MBGNs such as sol-gel and melt-derived approaches [14].

Conventionally applied melt-quenching and the sol-gel approaches can both be used to synthesize BG powders [15]. However, most melt-derived BGs suffer from several drawbacks. The oxide precursors may take up to 1500 °C for melting. Furthermore, studies suggest that only a narrow compositional range can offer the required bioactivity as silica concentrations above 60 mol.% render the material chemically inert in the presence of body fluids [3]. As a result of their superb bone-farming ability, predominant biocompatibility, and broadly ordered mesoporous structure, MBGNs play a vital role in BTE and controlled drug release [13].

Natural herbs have drawn attention for medicinal purposes due to their abundance and negligible side effects. In terms of personal tastes, the market for natural and organic products is growing, and the multiple benefits of herbal medications justify this form of treatment [16,17]. However, the range and restrictions of these medications must be determined by scientific investigations, which confirm the therapeutic effects. Therefore, a lot of researchers are interested in herb-loaded MBGNs for BTE applications [18,19].

Medical treatments made from plants are typically referred to as phototherapeutic entities. The utilization of phototherapeutic joined with BG is at the focal point of attention of numerous scientists to give elective, engineered, drug-loaded biomaterials for a diversity of operations, as reviewed by Refs. [20,21]. Among numerous therapeutic herbs, Peganum harmala (*P. harmala*; common name *harmala*) is traditionally used as a drug for pain relief and antiseptic effect. *P. harmala* is also said to prevent tumors, viruses, and fungi from spreading [22].

In this research, we concentrated on the development of *P. harmala-loaded* MBGNs (PH-MBGNs) for antibacterial purposes and *in-vitro* bioactivity. *P. harmala* was successfully loaded in MBGNs via the post-impregnation method.

To the authors’ best knowledge, PH-MBGNs have not been reported yet for BTE applications. The infrared spectroscopy using fourier transformation (FTIR) was used to confirm whether the *P. harmala* particles were loaded in MBGNs. PH-MBGNs formed a HA layer when soaked in the on-site prepared simulated body fluid (SBF). The sustained release of *P. harmala* from the PH-MBGNs inhibited the growth of gram-positive *(Staphylococcus aureus; S. aureus*) and gram-negative (*Escherichia coli; E. coli)* bacteria.

## 2 Materials and methods

### 2.1 Materials

Tetraethylorthosilicate (TEOS; Sigma Aldrich, Steinheim, Germany) of 99 % purity as Silica precursor and Calcium nitrate tetrahydrate (Ca(NO_3_)_2_.4H_2_O; Sigma Aldrich, Steinheim, Germany) of 98 % purity was used as calcium source. In addition, hexadecyltrimethylammonium bromide (CTAB: Merck, Billerica, MA, USA) of 98 %, purity, ammonium hydroxide (VWR, Shanghai, China) of 35 % purity, ethyl acetate (Sigma Aldrich, Germany) of 99.8 % purity, absolute ethanol of 99.8 % purity, and distilled water were used. *P. harmala* was procured from a local vendor in Pakistan. All synthetic substances utilized were of analytical grade. The reagents used for SBF preparation were purchased from VWR, Shanghai, China. Bacterial strains were collected from Islamabad Diagnostic Center (IDC) in Islamabad, Pakistan.

### 2.2 Synthesis of MBGNs

**Fig. 1:**
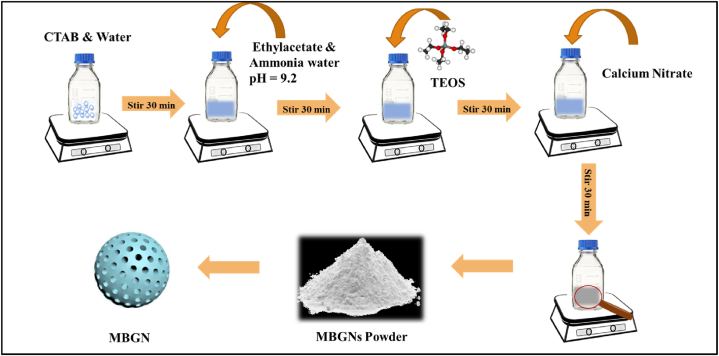
Synthesis of MBGNs by modified Stöber process.

The modified Stöber process was used to synthesize MBGNs [12]. The glass with 70 SiO_2_-30 CaO (mole %) composition was made. To begin, a solution of 0.56 g CTAB (soft template) in distilled water (26 mL) was prepared and stirred continuously at 30 °C for 30 min. Next, 8 mL ethyl acetate was added to the same mixture drop-wise. To keep the pH at 9.2, ammonium hydroxide (28 %) was added followed by TEOS (3 mL). The solution was stirred continuously. Finally, Ca (NO_3_)_2_.4H_2_O was added to the mixture and left for stirring for another 30 min. The solution was allowed to react for at least 3 h. The particles were separated and washed via centrifugation at 8000 rpm with distilled water and ethanol. The washing was done three times to remove any remnant chemicals. The particles were then dried for 12 h at 60 °C, then calcined for 5 h at 700 °C in air (Fig. 1) [23].

### 2.3 Extraction of P. Harmala

*P. harmala* roots were purchased locally (Pakistan), washed with distilled water, and ground to a fine powder after drying. A 10 mL solution of methanol and distilled water (8:2 v/v) was used to obtain the extract from powder (1 g). The supernatant was collected after 24 h by centrifuging the mixture for 15 min at 3000 rpm. Three times this technique was carried out. The solvent was then eliminated via evaporation [24].

### 2.4 Loading of P. harmala on MBGNS

**Fig. 2:**
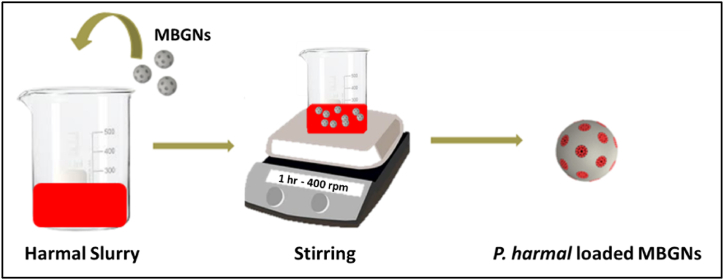
Loading of *P. harmala* on MBGNs.

Loading of *P. harmala* on MBGNs was done by magnetic stirring of 5 mg dried extract in 5 mL ethanol (10 % w/v) for 24 h. Then, the solution was ultrasonically treated for 5 min with 50 mg MBGNs. Later, the solution was stirred (400 rpm) for another hour at room temperature. In the end, the product was centrifuged at 2000 rpm and dried under ambient conditions (Fig. 2) [20].

### 2.5 Preparation of SBF

For SBF preparation according to Kokubo and Takadema’s recipe [25], glassware was cleaned with washing ethanol and dried in air. Deionized water (1.4 L) was measured and poured into a 2 L polyethylene bottle, which was then swirled with a magnetic stirrer. This bottle was then placed in a water bath. This whole assembly was heated on a hotplate to maintain a temperature of 36.5 ± 0.5 °C. Continuous stirring aids in maintaining temperature and water bath uniformity. After that, all reagents were weighed on a weighing balance with a precision of 0.001 mg and added one by one as listed in Table 1 until the previous one was fully dissolved. Agglomeration was avoided by constant stirring and moderate salt addition. Throughout the process, the temperature was kept at 36.5 ± 0.5 °C.

**Table 1:** Chemicals used for the synthesis of SBF [25].

Order	Reagent	Purity level	Amount in grams
1	Sodium Chloride (NaCl)	>99 %	16.1512
2	Sodium Bicarbonate (NaHCO_3_)	99.7 %	0.7065
3	Potassium Chloride (KCl)	99.7 %	0.4500
4	Potassium Phosphate Dibasic Trihydrate (K_2_HPO_4_.3H_2_0)	99.7 %	0.4620
5	Magnesium Chloride Hexahydrate (MgCl_2_.6H_2_0)	100.5 %	0.6065
6	Hydrochloric Acid (HCl)	1 mol/L	50 mL
7	Calcium Chloride Dihydrate (CaCl_2_.2H_2_O)	101 %	0.7275
8	Sodium Sulfate Anhydrous (Na_2_SO_4_)	99.6 %	0.1431
9	Tris (hydroxymethyl)aminomethane	100 %	12.1136
10	Hydrochloric Acid (HCl)	1 mol/L	20–30 mL

The pH of the mixture drops to <2 after 50 mL of HCl is added to it. The addition of tris increases the pH to 7.42–7.45 in the final stages. The pH changes between the two values until the whole tris is dissolved. To maintain a pH value of 7.4, 1 M HCl was added in the SBF. Total volume of the fluid was brought up to 2 L by adding de-ionized water at room temperature. This solution was kept in a cool place. It should be noted that if precipitates of salts settle down, the SBF cannot be used.

### 2.6 Characterization of PH-MBGNs

#### 2.6.1 Morphological and structural analysis

A scanning electron microscope (SEM: MIRA Tescan III) was employed to investigate the morphology of synthesized MBGNs. To prevent agglomerates, MBGNs were first dispersed in ethanol and then ultrasonically sonicated for 30 min. After drying on a glass slide, samples were sputtered with gold by a sputter coater to lessen the impact of charging. SEM was performed at an applied voltage ranging from 5 to 15 kV. Images were taken at various magnifications. The samples treated for bioactivity analysis were also observed under SEM. The elemental makeup of the samples was determined by energy dispersive x-ray spectroscopy (EDX) and presence of the various elements was assessed.

XRD (Mini Flex 600, Rigaku Corporation, Europe) was used to identify the structural nature of MBGNs such as amorphous or crystalline structure. The MBGNs were examined at a speed of 2°/minute while step size was kept at 0.02°. A 2ϑ range of 20°–80° was used.

#### 2.6.2 Fourier-transform infrared spectroscopy (FTIR)

The IR spectrum of transmission of material was obtained using the FTIR (Thermo-Scientific Nicolet Summit LITE). FTIR detects the chemical bonding of the material after different functional groups interact. FTIR was performed with wavelengths ranging from 400 to 4000 cm^−1^. Each sample was tested using 128 transmittance scans and a resolution of 4 cm^−1^.

#### 2.6.3 Brunauer-Emmett-Teller (BET) analysis

The nitrogen (N_2_) adsorption and desorption isotherm was obtained to compute the surface area of pure MBGNs and PH-MBGNs using the Brunauer-Emmett-Teller (BET) technique (GOLD APP Instruments, V-Sorb 2800, China). The porosity of MBGNs before and after loading P. harmala was determined using liquid N_2_. 20 mg of sample was degassed at 300 °C for 4 h before BET analysis.

#### 2.6.4 Surface charge and particle size distribution (PSD) measurement

Zeta potential was measured to determine the surface charge and PSD of nanoparticles via zetasizer (Malvern Zetasizer Nanozs90). This apparatus uses a combination of electrophoresis and Laser Doppler Velocimetry (LDV) to determine the zeta potential. In this technique, light is used that disperses over the sample at a 17° angle, causing the signal intensity to vary. Furthermore, the particle size distribution was acquired through Dynamic Light Scattering (DLS) technique. For reliable readings, the pure and PH-MBGNs mixtures in ethanol were diluted to 0.1 g/L.

#### 2.6.5 In-vitro drug release analysis

The quantification of loaded P. harmala was done by a UV–Vis spectrophotometer (GENESYS 10S) equipped with VISION lite Scan version 5.0 software at 208 nm 50 mg of PH-MBGNs powder was hydraulically pressed. The compressed pellets of 6 mm diameter were submerged in 10 mL of PBS. The samples were incubated in an orbital shaker (Biobase-BJPX-10-B) at 37 °C. At specified time intervals starting from 3 h, aliquots were taken out from PBS and the optical density (absorbance) of P. harmala was checked to assess the release of herb from MBGNs. The release was analyzed until no further absorbance was detected in the PBS. 2 mL solution was removed after each time point and refreshed with 2 mL PBS. The release study was conducted three times and average values with standard deviation were reported.

#### 2.6.6 Antibacterial behavior

To assess the bacterial resistance of particles, all the essential apparatus and nutrient agar were autoclaved for 15 min at 120 °C under 17 psi. Upon cooling, nutrient agar was poured into the plastic agar plates and incubated at 37 °C for 24 h. Bacterial strains were cultured by streak plate method, and plates were sealed and incubated for another 24 h.

Disk diffusion method: The antibacterial potential of the synthesized particles was first checked using disk diffusion technique. The minimum inhibitory concentration (MIC) was measured via this method. The pre-sterilized samples were incubated with 50 μL of *S. aureus* and *E. coli* spread on agar plates. The zone of inhibition was measured after 1 day.

Colony-forming unit (CFU): To further evaluate the antimicrobial effect, the CFU method was employed. The method is typically used to count the number of colonies on an agar plate after incubation. To investigate the antimicrobial activity of PH-MBGNs against bacterial strains*,* 0.05g PH-MBGNs powder was compressed into pellets (6 mm) form and sterilized under Ultraviolet (UV) light for 24 h before the test. After that, sterilized samples were added to 10 mL Nutrient broth with separately pre-cultured bacterial strains; *S. aureus* and *E. coli* and incubated at 37 °C for 48 h. Centrifugation of the suspensions was carried out at 11,000 rpm for 10 min. One mL supernatant from each suspension was transferred to sterilized tubes, inoculated with 5 μL of each bacterial strain, and incubated for another 24 h at 37 °C. Later, samples were serially diluted and an aliquot of 50 μL was spread on agar plates and again incubated under standard conditions. The total viable count was performed and CFU was determined.

#### 2.6.7 In-vitro bioactivity

The compressed pellets of PH-MBGNs were submerged in SBF to determine their in-vitro bioactivity. The samples (pellets with a diameter of 6 mm) were immersed in SBF medium (50 mL) for 21 days at 37 °C. SBF was refreshed after every 48 h to preserve the ionic concentration of the medium. The samples were taken out after 3, 7, and 21 days, rinsed with de-ionized water, and air-dried. SEM/EDX and FTIR were used to analyze the development of an appetite layer on the sample surface.

## 3 Results and discussion

### 3.1 Morphology and structure of the synthesized MBGNs

**Fig. 3:**
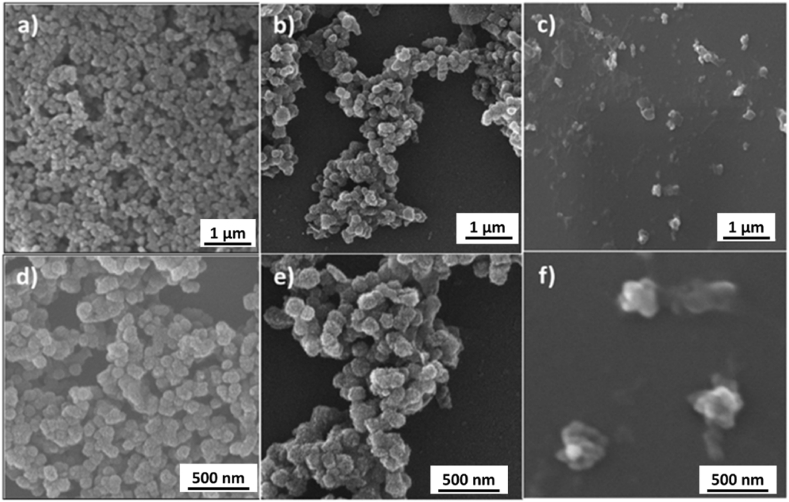
SEM image showing the surface morphology of a) MBGNs, b) PH-MBGNs, c) *P. harmala* at low magnification, and d) MBGNs e) PH-MBGNs f) *P. harmala* at higher magnification.

SEM was used to investigate the surface morphology of the synthesized MBGNs after loading *P. harmala* in them. SEM images of MBGNs and PH-MBGNs are shown in Fig. 3. SEM images indicated that the MBGNs were spherical and well dispersed with a uniform distribution in size. The average size calculated was 180 ± 15 nm. Images taken at high magnification revealed that particles had porous structure with irregular pores, as shown in Fig. 3 (a, d). The synthesized particles were similar in morphology to the ones reported by Nawaz et al. [10]. Moreover, Fig. 3 (b, e) shows SEM images of PH-MBGNs. While Fig. 3 (c, f) shows that particles of *P. harmala* are also in the nano range. The loading of *P. harmala* extract changed the morphology and particle size of MBGNs. This change in morphology and particle size may be attributed to the formation of surface layer upon loading the herb in MBGNs [20].

**Fig. 4:**
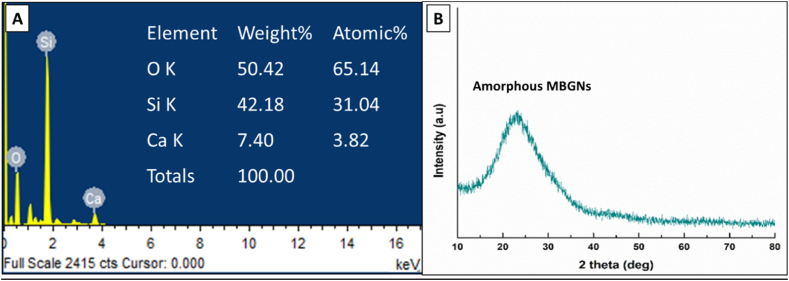
(A) EDX analysis of the synthesized MBGNs, (B) XRD of MBGNs depicting amorphous nature.

The composition of the sample surface was investigated using EDX. EDX confirmed the presence of Si and Ca in the MBGNs. Fig. 4A shows the elemental analysis of the MBGNs before *P. harmala* was added to them. The formation of MBGNs was affirmed via the detection of intense Ca and Si peaks in the spectrum [26].

XRD pattern of the MBGNs is shown in Fig. 4B. The appearance of broad peak in the pattern indicated that the composed MBGNs had an amorphous structure (broadband at 2θ ∼ 20°–32° exhibits typical amorphous properties of a glass). The similar amorphous nature of the bioactive glass particles was reported in Ref. [18].

### 3.2 FTIR analysis

**Fig. 5:**
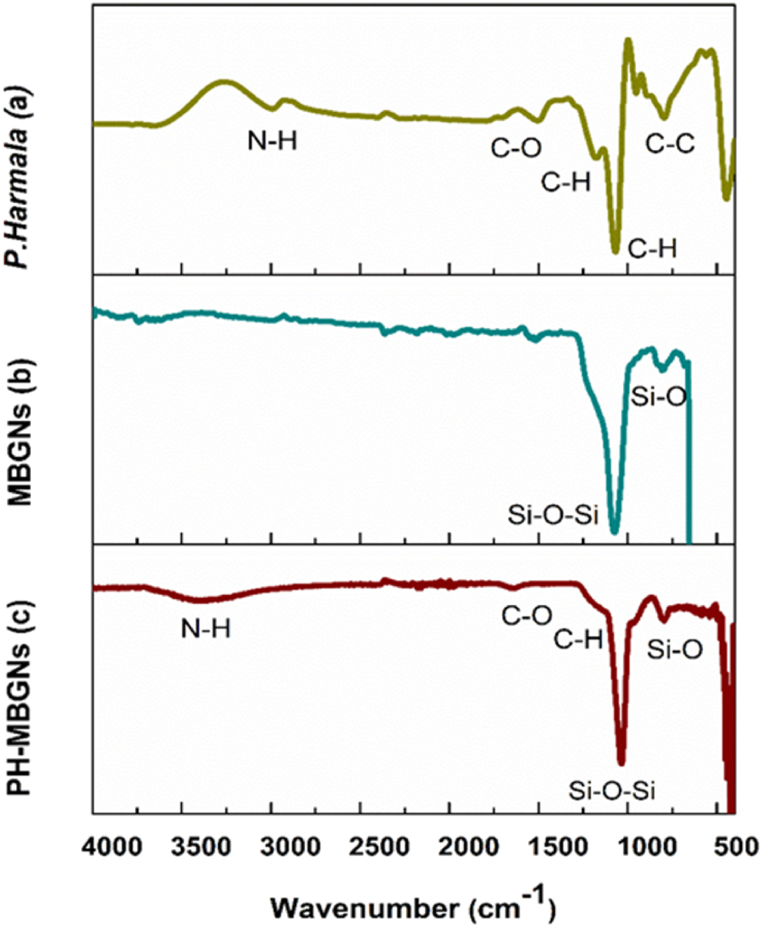
FTIR spectroscopy of (a) *P. harmala*, (b) MBGNs, and (c) PH-MBGNs.

The functional groups and chemical bonds in the MBGNs before and after loading *P. harmala* were determined using FTIR. Spectra of *P. harmala*, pure MBGNs, and PH-MBGNs are shown in Fig. 5. The identifying peaks of carboxylate stretching at 1192 cm^−1^ indicating C–H bonding and stretching at 1511 cm^−1^ of C–O bond are observed in Fig. 5a. Moreover, the peak at 2980 cm^−1^ corresponds to the amine group present in *P. harmala* [32].

The wide band detected at 1091 cm^− 1^ corresponds to stretching vibration of Si–O–Si as shown in Fig. 5 (b, c). The Si–O bonds’ wagging vibration was identified at 800 cm^−1^ [27]. Moreover, some of the peaks of *P. harmala* rearranged their position and potency after loading onto MBGNs (Fig. 5c), which is an indication of the established interaction of plant extract on MBGNs surface.

### 3.3 BET area examination

**Fig. 6:**
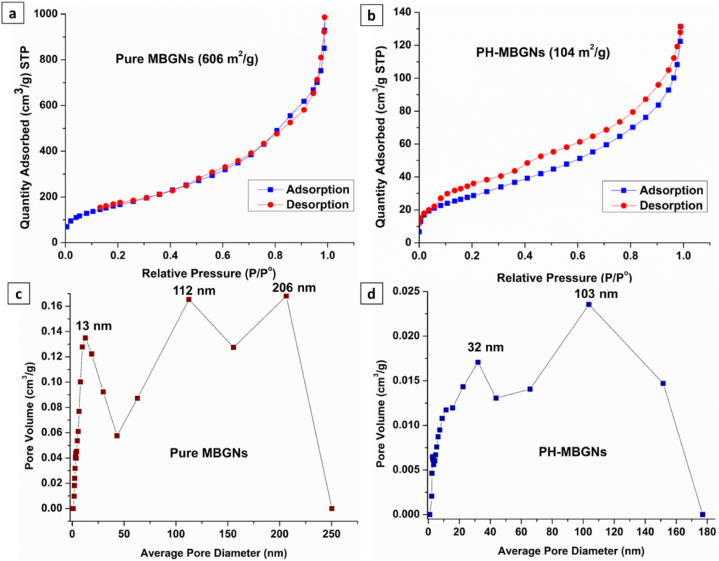
N_2_ isotherms representing (a) pure MBGNs and (b) PH-MBGNs. Porosity range of (c) pure MBGNs and (d) PH-MBGNs.

BET technique computed the surface area and porosity of the synthesized MBGNs and PH-MBGNs. Fig. 6 a,b shows the N_2_ adsorption-desorption isotherm in the form of IUPAC type IV (i.e. a hysteresis loop), demonstrating that the synthesized MBGNs contain mesopores; ranging from 2 nm to 50 nm [28]. An average surface area of 606 m^2^/g was calculated for MBGNs which decreased to 104 m^2^/g after drug loading. The pore size range was calculated using the BJH approach for MBGNs before and after loading *P. harmala*. The pore size curve in pure MBGNs (Fig. 6c) displayed three distributions. First was around 13 nm which confirmed the formation of mesopores in MBGNs, second and third distributions at 112 and 206 nm indicated that there were some macropores (>50 nm) also present in the MBGNs [28]. The drug-loaded MBGNs showed a slightly lowered pore size around 32 and 103 nm (Fig. 6d). It can be assumed that some particles of the drug were loaded in macropores of the MBGNs.

### 3.4 Zeta potential and PSD

Zeta potential is a simple way to determine the charge on the surface of MBGNs before and after herb loading. Table 2 shows the zeta potential and PSD of the MBGNs, *P. harmala*, and PH-MBGNs. This approach can also successfully identify any surface modification or surface charge change of MBGNs [25]. The surface of the MBGNs was found to have a negative zeta potential value of −10.7 ± 0.8 mV at a pH of 4 while *P. harmala* has a zeta potential of −8.30 ± 1.0 mV. On the other hand, PH-MBGNs show −8.20 ± 0.2 mV zeta potential that is very close to *P. harmala* indicating that MBGNs were loaded with *P. harmala* on the entire surface. The interaction within particles affected the PSD of nanoparticles as well. The size of MBGNs, *P. harmala,* and PH-MBGNs was measured using the DLS technique. MBGNs were approximately 190 ± 20 nm in size. After loading with *P. harmala* of average size 188 ± 12 nm, the size of the particle increased to approximately 295 ± 25 nm. The results of particle sizes observed in SEM and determined via zetasizer are in agreement with each other.

**Table 2:** Zeta potential and PSD of MBGNs before and after loading *P. harmala.*

Particles	Zeta Potential (mV)	PSD (nm)
MBGNs	−10.7 ± 0.8	190 ± 20
*P. harmala*	−8.30 ± 1.0	188 ± 12
PH-MBGNs	−8.20 ± 0.2	295 ± 25

### 3.5 In-vitro drug release studies

**Fig. 7:**
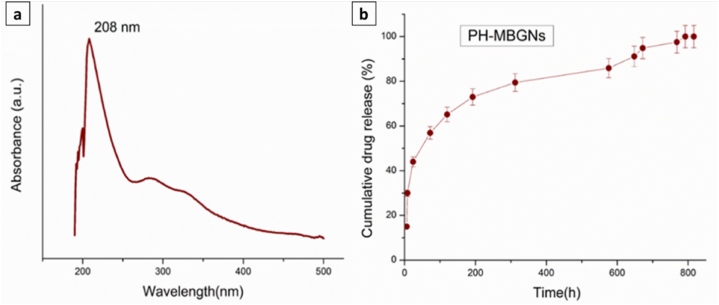
(a) Maximum absorbance (*λ*_max_) of *P. harmala,* (b) *In-vitro* release of *P. harmala* from PH-MBGNs in PBS.

To evaluate *P. harmala* release behavior from MBGNs, *in-vitro* drug release study was carried out with respect to time as shown in Fig. 7. At a wavelength of 208 nm, the maximum absorbance (max) of *P. harmala* was obtained (Fig. 7a). To calculate the released concentration, a calibration curve was drawn using known concentrations of *P. harmala*. Fig. 7b depicts the *P. harmala* release profile up to 810 h (∼34 days) in PBS. PH-MBGNs showed an initial burst release (approximately 30% in 8 h), followed by a slow and steady release till 810 h. It is suggested that some of *P. harmala* particles adhering to the MBGNs' surface diffused fast into the solution and resulted in the preliminary burst release [25]. Following that, a sustained release from PH-MBGNs was noticed. The possible reasons for the regulated release could be that most of the *P. harmala* particles were electrostatically attached to the surface of the MBGNs or collected in the channels of mesopores [31]. The total release of *P. harmala* from the MBGNs approached 99.9 % of the total loaded amount after 810 h.

### 3.6 Antibacterial analyses

**Fig. 8:**
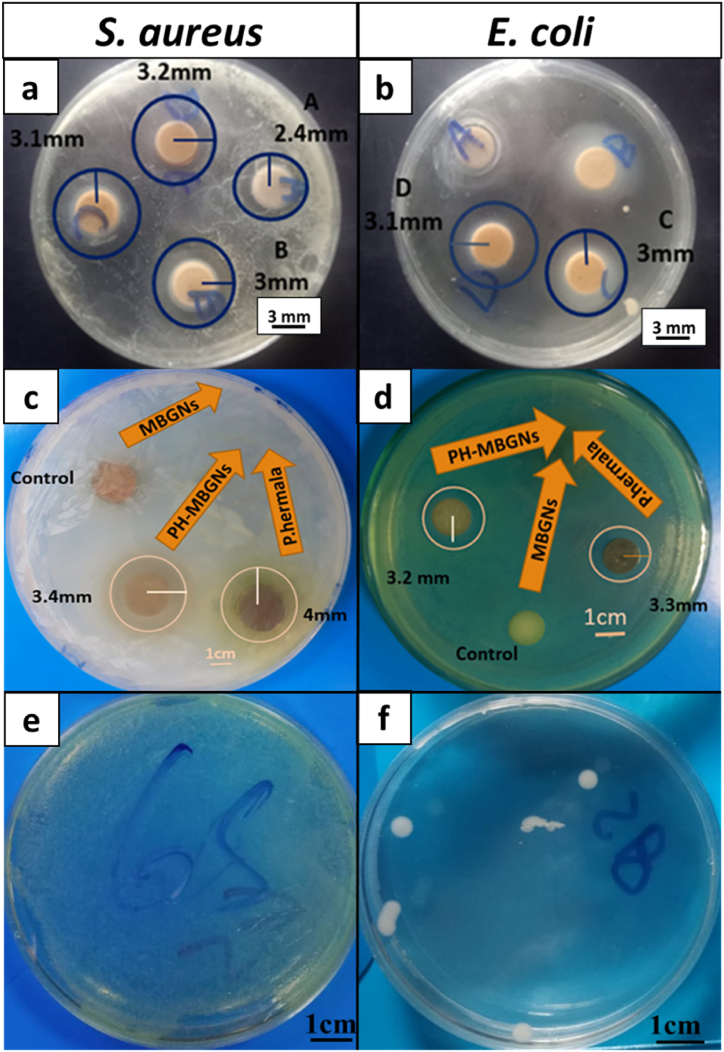
MIC (a, b), antibacterial activity (c, d), and CFU (e, f) results of *P. harmala* against *S. aureus* (a, c, e) and *E. coli* (b, d, f).

The bacterial resistance of PH-MBGNs was assessed by halo test. As reported, *P. harmala* has a toxicity of 50 mg/kg [29]. MIC was performed to determine the minimum effective dose for antibacterial activity of PH-MBGNs. MBGNs were loaded with various amounts of *P. harmala,* as shown in Table 3 for this purpose. Fig. 8 shows the MIC test results against *S. aureus* and *E. coli* (Fig. 8 a,b) after 24 h of incubation. Samples A and B with a lower concentration of herb did not show a substantial inhibitory zone for *E. coli* but showed a considerable impact on *S. aureus*. A 2.4 mm wide zone of inhibition was observed for *S. aureus* at the lowest concentration of PH-MBGNs. On the other hand, there was negligible reduction in the growth of *E. coli* for it showed a very small inhibition zone. Against sample C, both bacteria showed a significant inhibition zone. As a result, we proceeded with sample C for further research (10 w/w %).

**Table 3:** Concentration of PH-MBGNs for MIC test.

Name of Sample	PH-MBGNs (w/w %)
*A*	*2*
*B*	*6*
*C*	*10*
*D*	*14*

After determining the MIC for effective bacterial control, the antibacterial behavior of pure MBGNs*, P. harmala,* and PH-MBGNs (with sample C concentration) were assessed using the same method. It was revealed that pure MBGNs did not show any significant control on bacterial growth Fig. 8 (c, d). However, the antibacterial capabilities were dramatically improved after adding plant extract to MBGNs against both strains. Immense research on the antibacterial potential of extracts collected from various *P. harmala* species, concluded that the extracts showed superior resistance to gram-positive bacteria whereas a slight resistance to the growth of gram-negative bacteria [[30], [31], [32]].

The biologically active components of *P. harmala*, such as the main alkaloids harmaline, harmane, harmalol, and peganine, which have the potential to intercalate with the DNA of microbes, could be the reason for the antibacterial effect [29,33]. According to reports, harmane, a highly aromatic planar alkaloid, acts as an antibacterial agent by intercalating with DNA [34]. The high concentration of polyphenols, with known antibacterial activity, may potentially be responsible for *P. harmala's* antibacterial effect. Aqueous and alcoholic extracts of *P. harmala* repressed the development of *Candida albicans* and *Lactobacilli* regularly tracked down in the oral cavity [31,35]. Conversely, the inherent exterior lipophilic membrane of *E. coli* can be used to explain why the PH-MBGNs have reduced antibacterial efficacy against gram-negative bacteria. Lipopolysaccharide molecules dominate this outer layer, forming a hydrophilic permeability barrier that guards against the impacts of very hydrophobic substances [36].

Furthermore, the bacterial resistance of particles was confirmed by the CFU method (Fig. 8f, e). *E. coli* and *S. aureus* cultured in nutrient broth were allowed to react with PH-MBGNs at 37 °C for 48 h. Later on, the cultures were serially diluted and spread over nutrient agar plates and after an incubation time of 24 h, a colony count was performed. In the present study, complete inhibition of *S. aureus* from all the incubated plates was observed whereas partial inhibition was observed against *E. coli*. Hence, we were able to confirm the antimicrobial effect of PH-MBGNs due the release of *P. harmala* compound from MBGNs [32].

### 3.7 In-vitro bioactivity in SBF

**Fig. 9:**
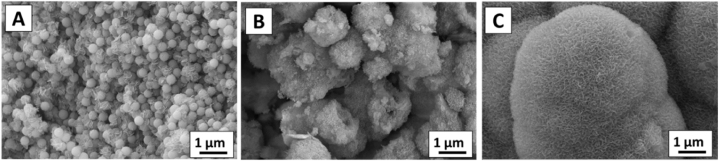
*In-vitro* bioactivity of PH-MBGNs, (A) nucleation of HA starts at day 3, (B) HA layer spreading on day 7, (C) full coverage of HA at day 21.

The influence of particle bioactivity (defined as a material's ability to produce HA on its surface) was investigated by soaking the pellets for 21 days in SBF; an acellular solution containing the inorganic components of blood plasma (Fig. 9). The apatite layer started to form on PH-MBGNs on day 3, as shown in Fig. 9A. The HA crystals were more visible on PH-MBGNs’ surface on day 7 (Fig. 9B). After that, a three-dimensional layer entirely covered the surface of PH-MBGNs, as shown in Fig. 9C [37]. On the surface of the pellet, the apatite layer was dense and thick, with some precipitation inside the pores. The inclusion of *P. harmala* greatly boosted bioactivity, as stated in the literature [37]. It’s worth noting that PH-MBGNs displayed bioactivity after only 3 days of incubation and continued to be bioactive until 21 days. The bioactivity (*in-vitro*) of pure MBGNs in SBF was also presented in our previous article [14].

**Fig. 10:**
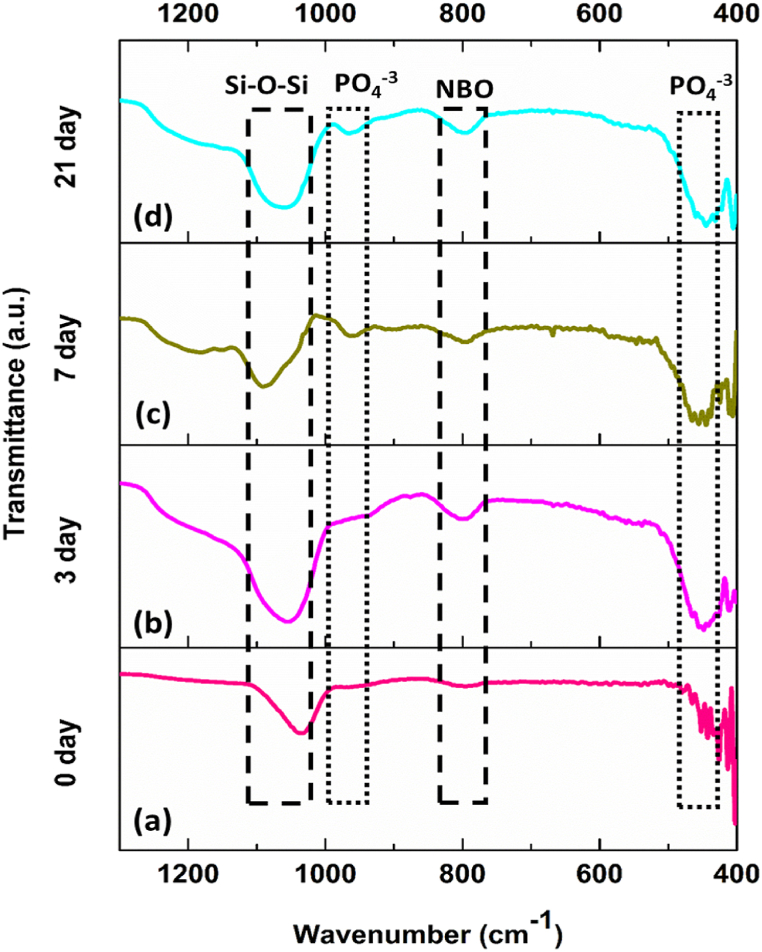
FTIR of PH-MBGNs samples after immersion in SBF at specified days.

The bioactive; HA layer formation was further confirmed by employing FTIR. The spectra of PH-MBGNs before (0 days) and after treating with SBF (for 3, 7, and 21 days) were recorded (Fig. 10). The peaks indicating any change in the MBGNs structure due to their bioactive nature were detected between 400 and 1200 cm^−1^. At 1050 cm^−1^, peak of Si–O–Si bond was prominent in the un-immersed sample (Fig. 10a). After 3 days in SBF, particles started to show bioactivity, as evidenced by the presence of a slight curve at 963 cm^−1^ related to the stretching of PO_4_^−3^ bonds in HA (Fig. 10b) [38]. The peak at 963 cm^−1^ further became intense on day 7 as indicated in Fig. 10c. On day 21, the Si–O–Si bond became broader as compared to the earlier days (Fig. 10d), indicating that the HA layer started forming and the MBGNs network started to disrupt. Furthermore, amorphous phosphate was represented by a band in the 450–470 cm^−1^ range [39] which also became prominent at day 3 in SBF. The peak observed around 800 cm^−1^ (on day 3 and after) corresponded to the non-bridging oxygen of the silica network of bioactive glass [40]. This peak was an indicator of the disturbance in the silica network due to the interaction of MBGNs with SBF as time passes.

## 4 Conclusions

We opted for a facile methodology to load the therapeutic agent *P. harmala* on the MBGNs in this study. We developed and characterized PH-MBGNs with antibacterial and bioactive characteristics. These objectives were achieved by loading *P. harmala* on the MBGNs. Spherically shaped carrier particles of 180 nm were successfully synthesized using a modified Stöber process and post-modification procedure. SEM-EDX analyses evaluated desired morphological properties and the presence of all desired components in PH-MBGNs. The amorphous structure of MBGNs was confirmed by a broad peak in XRD.

FTIR and zeta potential showed attachment of *P. harmala* to the BG surface. The bioactivity was assessed utilizing SBF, which indicated the formation of a HA layer, while FTIR analysis revealed an increase in P content with time. Furthermore, BET analysis revealed a larger surface area and mesoporous structure of MBGNs that can be used for biomedical applications because of their capability of drug loading. PH-MBGNs effectively resisted the growth of bacteria; *S. aureus* and *E. coli*. In conclusion, PH-MBGNs may be an interesting material for biomedical applications such as BTE.

## Data availability statement

No data was used for the research described in the article.

## Funding

The authors extend their appreciation to the Deanship of Scientific Research at 10.13039/501100007446King Khalid University (KKU) for funding this research through the Research Group Program Under the Grant Number:(R.G.P.1/429/44).

## CRediT authorship contribution statement

**Maria Bibi:** Investigation, Methodology, Writing – original draft. **Syeda Ammara Batool:** Conceptualization, Investigation, Methodology, Visualization, Writing – original draft. **Sajid Iqbal:** Investigation, Validation, Writing – original draft. **Shaher Bano Zaidi:** Investigation, Methodology, Writing – original draft. **Rabia Hussain:** Formal analysis, Methodology, Resources, Writing – original draft, Writing – review & editing. **Memoona Akhtar:** Investigation, Supervision, Visualization, Writing – review & editing. **Ahmad Khan:** Formal analysis, Investigation, Methodology, Validation. **Mohammed S. Alqahtani:** Funding acquisition, Project administration, Supervision, Validation, Writing – review & editing. **Mohamed Abbas:** Funding acquisition, Supervision, Validation, Writing – review & editing. **Muhammad Atiq Ur Rehman:** Conceptualization, Methodology, Project administration, Supervision, Validation, Visualization, Writing – original draft, Writing – review & editing.

## Declaration of competing interest

The authors declare that they have no known competing financial interests or personal relationships that could have appeared to influence the work reported in this paper.
